# Source-tracking of the Chinese Chikungunya viruses suggests that Indian subcontinent and Southeast Asia act as major hubs for the recent global spread of Chikungunya virus

**DOI:** 10.1186/s12985-021-01665-2

**Published:** 2021-10-11

**Authors:** Shaofu Qiu, Jinpeng Guo, Peihan Li, Peng Li, Xinying Du, Rongzhang Hao, Chaojie Yang, Qi Wang, Hongbo Liu, Haoran Zhang, Sai Tian, Hua Shi, Liang Wen, Daizhi An, Xiaocui Yang, Xiaoyuan An, Ligui Wang, Changjun Wang, Hongbin Song

**Affiliations:** 1 The Chinese PLA Center for Disease Control and Prevention, 20 DongDa Street, Fengtai District, Beijing, 100071 China; 2 The Fourth People’s Hospital of Zunyi, Guizhou, China

**Keywords:** Chikungunya virus, Whole-genome sequencing, Phylogenetic analysis, Evolution, Adaptive mutation

## Abstract

**Background:**

Chikungunya fever, caused by the Chikungunya virus (CHIKV), has become a major global health concern, causing unexpected large outbreaks in Africa, Asia, Europe, and the Americas. CHIKV is not indigenous to China, and its origin in the country is poorly understood. In particular, there is limited understanding of the recent global spread of CHIKV in the context of the CHIKV epidemic.

**Methods:**

Here we investigated a novel Chikungunya patient who came from Myanmar to China in August, 2019. Direct genome sequencing was performed via combined MinION sequencing and BGISEQ-500 sequencing. A complete CHIKV genome dataset, including 727 CHIKV genomes retrieved from GenBank and the genome sequenced in this study, was constructed. An updated and comprehensive phylogenetic analysis was conducted to understand the virus’s origin, evolution, transmission routes and genetic adaptation.

**Results:**

All globally distributed CHIKV genomes were divided into West Africa, East/Central/South African and Asian genotypes. The genome sequenced in this study was located in the Indian Ocean lineage, and was closely related to a strain isolated from an Australian patient who returned from Bangladesh in 2017. A comprehensive phylogenetic analysis showed that the Chinese strains mainly originated from the Indian subcontinent and Southeast Asia. Further analyses indicated that the Indian subcontinent and Southeast Asia may act as major hubs for the recent global spread of CHIKV, leading to multiple outbreaks and epidemics. Moreover, we identified 179 distinct sites, including some undescribed sites in the structural and non-structural proteins, which exhibited apparent genetic variations associated with different CHIKV lineages.

**Conclusions:**

Here we report a novel CHIKV isolate from a chikungunya patient who came from Myanmar to China in 2019, and summarize the source and evolution of Chinese CHIKV strains. Our present findings provide a better understanding of the recent global evolution of CHIKV, highlighting the urgent need for strengthened surveillance against viral diversity.

**Supplementary Information:**

The online version contains supplementary material available at 10.1186/s12985-021-01665-2.

## Introduction

The Chikungunya virus (CHIKV) causes chikungunya fever, a febrile illness with severe arthralgia and rash, and even severe clinical symptoms [1]. CHIKV reportedly originated from Africa, and the first case of chikungunya fever was recognized in an outbreak in Tanzania in 1952 [2]. Since then, CHIKV has caused multiple outbreaks and epidemics [1, 3]. Since 2004, CHIKV has caused unexpected large outbreaks in Africa, Asia, Europe, and the Americas, becoming a major global health concern [3, 4].

Uncovering the epidemiological patterns and dynamic trends of CHIKV is crucial for its prevention and control. China is a non-endemic region of CHIKV, and most cases were imported and identified in the Guangdong and Zhejiang Provinces [2, 4]. Several local chikungunya outbreaks have also been documented in these two Provinces [2, 4]. Here we identified a novel CHIKV isolate from a chikungunya patient who came from Myanmar in August, 2019. The origin of Chinese CHIKV strains is poorly understood. Previous studies have described the origin, evolution and spread of CHIKV globally. However, there is limited understanding of the recent global spread of CHIKV in the context of CHIKV epidemic. Here an updated and comprehensive phylogenetic analysis was conducted by using a global genome dataset to understand the virus's origin, evolution, transmission routes and genetic adaptation.

## Methods

A 42-year-old Chinese male patient with fever, cough and fatigue was admitted to a hospital in Zunyi City, Guizhou Province, China, on August 8, 2019. In 2019, this patient was engaged in pepper planting and sales in Yangon, Myanmar. He had an abrupt fever at about 17:00 on August 7, 2019. He then flew back to Kunming City, Yunnan Province, China at 19:00, and returned to Zunyi City for emergency treatment at 6:00 on August 8, 2019. Blood samples were collected and sent to our laboratory for further microbiological detection. A multiplex quantitative reverse transcription-PCR (qRT-PCR) kit (Shenzhen Uni-medica Co.,Ltd, Shenzhen, China) was used to screen for seven pathogens including CHIKV, Dengue, Japanese encephalitis, West Nile, Yellow fever, Sindbis and Zika viruses. The patient was given supportive treatment, including Cefuroxime sodium, Qingkailing injection, and Vitamin C injection, according to prescribed medications. After 11 days of treatment, the patient recovered and was discharged from the hospital.

Direct whole-genome sequencing (WGS) was performed using the MinION and BGISEQ-500 platforms. De novo assemblies were generated using Canu v1.6 [5] for MinION sequencing and MEGAHIT V1.1.4 [6] for BGISEQ-500 sequencing. We constructed a complete CHIKV genome dataset, including 727 CHIKV genomes retrieved from Genbank (access date 11/01/2019) and the genome ZY1908 (accession no. MN756625) sequenced in this study (Additional file 1: Table S1). A comprehensive phylogenetic analysis was performed to trace the origin of the Chinese strains and to understand the origin, evolution, and transmission routes of the virus. To better understand the evolutionary adaptations of the CHIKV genotypes, a thorough screening of the lineage-specific varieties in structural and non-structural proteins (sP and nsP), was conducted based on the 728 CHIKV sequences.

## Results

**Fig. 1:**
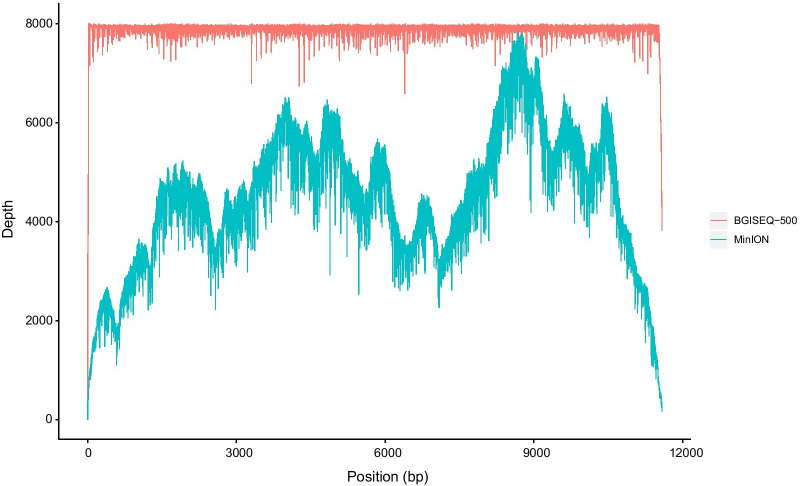
Coverage depth across the chikungunya viral genome using MinION and BGISEQ-500 platforms. Coverage depth is shown in blue and red for MinION and BGISEQ-500 platforms, respectively

**Fig. 2:**
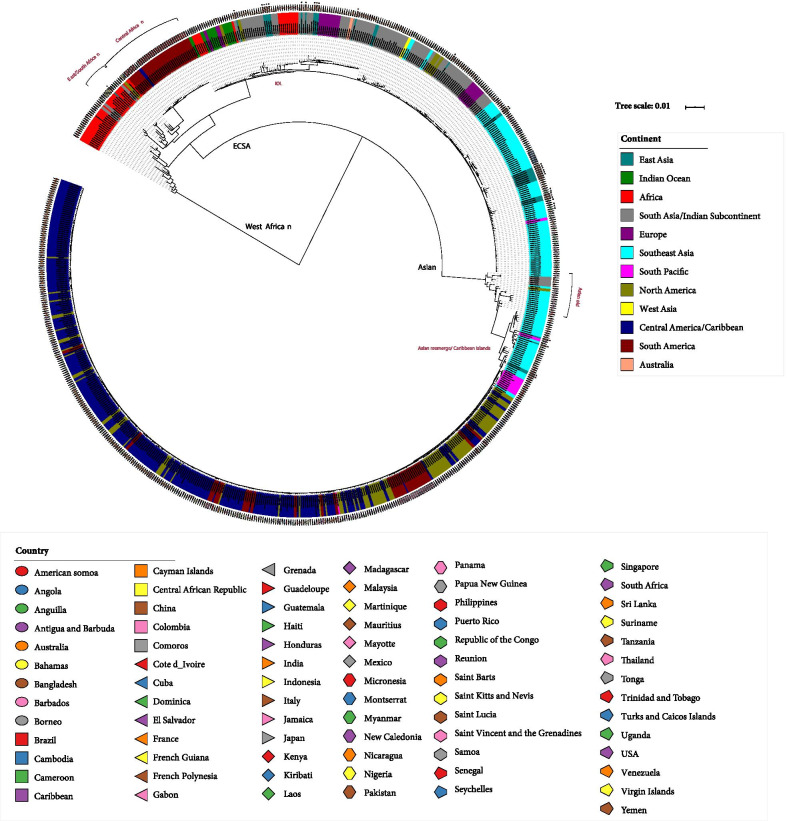
Phylogenetic tree constructed based on the maximum likelihood method by using an updated CHIKV genome dataset. A total of 728 genomes are used, including 727 genomes newly retrieved from GenBank and the genome (ZY0814) sequenced in this study. Strains are named by GenBank accession number, sample location and year of isolation, and are color-coded for sample location including continents (inner circle) or countries (outer circle). The Chinese and Myanmar strains are highlighted in red dots and green triangles, respectively. CHIKV, chikungunya virus; ECSA, East/Central/South African; IOL, Indian Ocean lineage

Epidemiological investigation showed that this patient resided in Yangon, Myanmar for nearly half a year. He had been bitten by mosquitoes on his calf before the onset of the disease. The qRT-PCR result showed that chikungunya virus was positive, but other pathogens were negative. The genome was rapidly and directly obtained from the serum sample via combined MinION and BGISEQ-500 sequencing (Fig. 1). Here we obtained 29 Chinese CHIKV genomes from GenBank (Additional file 2: Table S2). Phylogenetic analysis showed that most (79.3%) Chinese genomes were located in the Indian Ocean lineage (IOL) of the East/Central/South African (ECSA) genotype, and clustered with the Indian strains(Fig. 2). Some strains were located in the Asian genotype and were closely related to strains from Indonesia and Philippines. The genome sequenced in this study was located in the IOL lineage (Fig. 2), and was closely related to a strain (accession no. MF773566) isolated from a patient in Australia who returned from Bangladesh in 2017 and to two Chinese strains (Accession Nos. MG912993 and MH400249) isolated from a local chikungunya outbreak that occurred in the Zhejiang Province in 2017 [4].

The globally distributed CHIKV genomes were divided into West Africa (WA), ECSA and Asian genotypes (Fig. 2). Most genomes under the ECSA genotype (80.9%) were clustered in the IOL lineage, which probably originated from Kenya. This spread along the Indian Ocean to Comoros, La Reunion, Mauritius, Mayotte, Sri Lanka and India. From the Indian Subcontinent, it spread to Southeast Asia and Europe (Fig. 2). Most strains under the Asian genotype were isolated from the Americas (84.9%), and developed into a new sublineage named Asian reemerge/Caribbean Islands. This sublineage likely originated from Southeast Asia, particularly Thailand. It then spread along the South Pacific to Federated States of Micronesia, Tonga, Samoa, American Samoa, Kiribati, and then to the Americas, including the Caribbean, United States, Colombia, Dominica, Mexico, Nicaragua, Haiti and so on (Fig. 2).

**Fig. 3:**
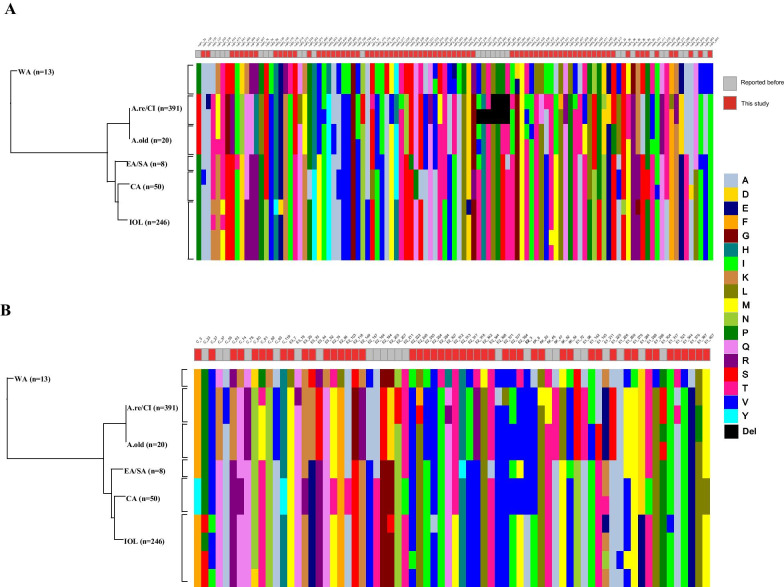
The lineage-specific genetic varieties in non-structural and structural proteins of the CHIKV strains. Alignments of the amino acid sequences were performed to determine the consensus sequence. The consensus sequences from each genotype/lineage were aligned to screen the lineage-specific varieties. The amino acid positions with a difference of more than 1% were selected and the sites unrelated to lineages were removed. The lineage-specific genetic varieties in non-structural and structural proteins of the CHIKV strains are shown in panel **a** and **b**, respectively, with 72 and 107 mutational sites. The amino acid varieties in sites are represented by squares in different colors. The amino acid positions in each viral protein are listed by corresponding numbers. The undescribed mutational sites are marked in red squares and those previously described are marked in grey squares. CHIKV, chikungunya virus; WA, West African; A.re/CI, Asian reemerge/Caribbean Islands; A.old, Asian old; EA/SA, East/South African; CA, Central African; IOL, Indian Ocean lineage

Subsequently, the detailed genetic varieties of the viral proteins of the CHIKV lineages were characterized. A total of 179 distinct sites, including 52 previously described sites and 127 undescribed sites, were identifiedin the sP and nsP proteins (Fig. 3 and Additional file 3: Table S3). Among the undescribed sites, 78 were identified in the nsP proteins and 49 in the sP proteins. There were 72 and 107 mutational sites identified in the sP and nsP proteins, respectively. Among the mutational sites identified in the nsP proteins, most (47.7%) were observed in the nsP3 protein, and E2 protein was determined to be among the mutation hotspots for the sP proteins. They exhibited apparent genetic variations associated with different CHIKV lineages (Fig. 3). For example, some distinct sites in the nsP proteins (nsP1-K488R, nsP2-S54N, nsP2-A793V, nsP3-T337I, nsP3-P471S, and nsP4-T254A), as well as some in the sP proteins (E2-T312M, 6K-A8I, and E1-D284E) were observed in IOL lineage.

## Discussion

In this study we report a novel chikungunya case imported from Myanmar to China. At least four other cases involved patients traveling from Myanmar to China in 2019 (http://www.chinanews.com/sh/2019/06-28/8877963.shtml; https://www.cn-healthcare.com/article/20191106/content-525783. html). Four chikungunya cases were identified in Myanmyar in 2010. However, there were no reported casesduring 2011–2018 [7]. In 2019, health officials in Myanmar had reported a significant increase in chikungunya cases (https://www.mmtimes.com/news/chikungunya-reappears-after-10-years.html). This changing trend indicates the potential reemergence and epidemic of CHIKV in Myanmar.

To determine the origin of the Chinese CHIKV strains, a comprehensive phylogenetic analysis was performed. The Chinese strains mainly originated from the Indian Subcontinent and Southeast Asia, suggesting a potential role of the Indian Subcontinent and Southeast Asia in promoting the global spread of CHIKV. Then we investigated and observed that two main genotypes probably mediated the recent global spread of CHIKV. The recent emergence and epidemic of the ECSA genotype were first recognized in an outbreak in Kenya in 2004. Following rapid transmission to the Indian Ocean islands and the Indian Subcontinent, it was named the IOL lineage [8]. Phylogenetic analysis revealed the possible transmission route of the IOL lineage, suggesting that the Indian Subcontinent especially India, was a hub for its rapid global dissemination. Currently, the IOL lineage has caused a series of explosive outbreaks, involving millions of people in the Indian Subcontinent, Southeast Asia and Europe [2, 3, 9]. Other recent episodes of CHIKV reemergence included the worldwide endemic Asian genotype, which has developed into a new Asian reemerge/Caribbean Islands sublineage. Phylogenetic analysis showed that this sublineage likely originated from Southeast Asia, and is now endemic to the Americas. The first autochthonous transmission of the Asian genotype in the Americas was reported in the Caribbean island in 2013. Since then, the Caribbean region may has become a major dispersal focus for the subsequent dissemination of this new sublineage, which has caused a large-scale epidemic, involving almost 45 countries and territories in North, Central and South America [8, 10]. Based on the analyses, the recent emergent lineages displayed different evolutionary paths. The Indian subcontinent and Southeast Asian regions possibly acted as major hubs for the recent global spread of different CHIKV genotypes.

Previous studies have shown that genetic mutations in CHIKV are important in its adaptation to new vectors or the host immune system [11, 12]. Some mutations at key sites of CHIKV E1 and E2 genes are involved in the virus adaption in the local mosquito species and thus influenced the spatial arrangement of different strains [13]. Virus containing E1-A226V and E2-I211T mutations showed enhanced infectivity of CHIKV in *Ae*. *albopictus* [14]. The adaptive mutations in the E1 (A226V) and E2 (K252Q) genes in the IOL lineage were reportedly associated with an increased viral fitness towards *Ae. albopictus* [12]. Two specific mutations in E2 (V368A) and 6K (L20M) genes were found in the Caribbean clade, possibly associated with the increased viral fitness and replication towards *Ae. aegypti* [10]. Moreover, a deletion, nsP3 deletion 379–382 was observed in A.re/CI genotype. This deletion appears to be host and virus specific, which confers the ability to infect *Anopheles* mosquitoes [11]. Surprisingly, here we identified a series of lineage-specific mutations in the sP and nsP proteins, including some undescribed amino acid sites. The mutational hotspots in the sP and nsP genes identified in this study will be useful in determining the viral fitness and tracking the source of the virus.

The cocirculation of multiple lineages were observed in some regions, especially Southeast Asia, Indian subcontinent and Americas. The cocirculation of the Asian and ECSA lineages has occurred in Brazil since 2014, while the cocirculation of the Asian and IOL lineages was observed in USA [11]. The cocirculation of the IOL and Asian lineages was more complicated in Southeast Asia, where cocirculation has been identified for years [3]. With increasing globalization, climate change, continuous dispersal of *Aedes* mosquitoes, and adaptive viral mutations, CHIKV will continue to expand its global distribution and cause the potential cocirculation of diverse genotypes, becoming an increasing threat to public health.

## Conclusions

In conclusion, here we reported a novel CHIKV isolate from a Chikungunya patient who came from Myanmar to China in 2019. The genome was directly obtained from the serum sample by combined MinION sequencing and BGISEQ-500 sequencing. Phylogenetic analysis showed that the genome sequenced in this study was located in the IOL lineage, and the Chinese strains mainly originated from the Indian subcontinent and Southeast Asia, implying the potential role of these regions in CHIKV transmission. Further analyses indicated that the Indian subcontinent and Southeast Asia acted as major hubs for the current global spread of CHIKV, leading to multiple epidemics and outbreaks. In addition, a series of lineage-specific adaptive mutations possibly associated with vector preference and different transmission routes were identified. The present study provides a better understanding of the recent global evolution of CHIKV, suggesting that CHIKV is an increasing threat to public health. It highlights the urgent need for strengthened surveillance against viral diversity.

## Supplementary Information

**Additional file 1: Table S1**. The CHIKV genomes used in this study.

**Additional file 2: Table S2**. Summary of the Chinese CHIKV strains with genome sequences.

**Additional file 3: Table S3**. The distinct mutations associated with CHIKV genotype/lineages.

## Data Availability

The genome sequenced in this study was deposited in GenBank under the Accession Number MN756625, and the data analyzed during this study are included in Additional file 1: Table S1.

## Abbreviations

CHIKV: Chikungunya virus
WGS: Whole-genome sequencing
ECSA: East/Central/South African
IOL: Indian Ocean lineage
